# Use frequency of traditional Chinese medicine in Taiwan

**DOI:** 10.1186/1472-6963-7-26

**Published:** 2007-02-23

**Authors:** Fang-Pey Chen, Tzeng-Ji Chen, Yen-Ying Kung, Yu-Chun Chen, Li-Fang Chou, Fan-Jou Chen, Shinn-Jang Hwang

**Affiliations:** 1 Center for Traditional Medicine, Taipei Veterans General Hospital, No.201, Section 2, Shih-Pai Road, Taipei 112 Taiwan; 2 National Yang-Ming University School of Medicine, No.155, Section 2, Linong Street, Peitou District, Taipei 112, Taiwan; 3 Department of Family Medicine, Taipei Veterans General Hospital, No.201, Section 2, Shih-Pai Road, Taipei 112 Taiwan; 4 Department of Public Finance, National ChengChi University, No. 64, Section 2, Zhinan Road, Wenshan District, Taipei 116 Taiwan; 5 Graduate Institute of Integration Chinese and Western Medicine, Chinese Medical University, No. 91, Hsueh-Shih Road, Taichung 404 Taiwan

## Abstract

**Background:**

Use of Traditional Chinese medicine (TCM), an important category of complementary and alternative medicine (CAM), has increased substantially in Western countries during the past decade. Use of TCM is also widespread in the Chinese population. However, few informative data have been obtained to date by large-scale investigations of TCM use in the Chinese population. This study was aimed at elucidating the demographics and patterns of TCM use in Taiwan.

**Methods:**

We employed the complete datasets of TCM outpatient reimbursement claims from 1996 to 2001, including the use of Chinese herbal remedies, acupuncture and traumatology manipulative therapy, to analyse use frequencies, the characteristics of TCM users, and the disease categories that were treated by TCM in Taiwan.

**Results:**

At the end of 2001, 6,142,829 (28.4%) among the 21,653,555 valid beneficiaries of the National Health Insurance in Taiwan had used TCM during the year. However, 13,536,266 subjects (62.5%) had used TCM at least once during the whole 6-year period from 1996 to 2001, with a total of 156,224,266 visits (mean 11.5 visits per user). The mean number of TCM users per annum was 5,733,602, with a mean increment of 1,671,476 (29.2%) of new users yearly. Among TCM users, female was higher than male (female:male = 1.13:1), and the age distribution displayed a peak at around the 30s, followed by the 20s and 40s. Chinese herbal remedies (85.9%) were the most common TCM modality used by this population, followed by acupuncture (11.0%) and traumatology manipulative therapies (3.1%). Private TCM clinics provided most of the TCM care (82.6%), followed by private TCM hospitals (12.0%). The top ten major disease categories for TCM visits were diseases of the respiratory system, musculoskeletal system and connective tissue; symptoms, signs and ill-defined conditions; injury and poisoning; diseases of the digestive system, genitourinary system, skin and subcutaneous tissue, nervous system and sense organs, circulatory and endocrine system; nutritional and metabolic diseases; and immunological disorders.

**Conclusion:**

TCM was popular among the Chinese population in Taiwan during the period studied. More than 60% of all subjects had used TCM during the 6-year interval. TCM was widely used by the Chinese population to treat problems and diseases of major human organ systems recognised by western medicine. This study provides information about the use frequencies of TCM and the disease categories treated by TCM, which should be useful for health policy makers and for those considering the integration of TCM and Western medicine.

## Background

Interest in complementary and alternative medicine (CAM) has increased substantially in western countries during the past decade [1-4]. Patients and their families seem to have sought their health practitioners' opinions about various CAM modalities more frequently [1,2]. Recent studies have demonstrated dramatic increases in the use of, and expenditure on, CAM in the United States, Canada, Australia and European countries [5-8]. However, most of the prevalent studies of CAM use were based primarily on questionnaire surveys, telephone interviews or collecting data from insurance claims, and the sample sizes generally were limited.

Traditional Chinese medicine (TCM) is an important category of CAM in Western opinion [9]. Current TCM practices can be traced back more than 2000 years. The concepts of Ying-Yang, Five Elements, Meridians, and the use of many herbal remedies, originated from ancient China [10,11]. TCM is still commonly used by the Chinese [4,12,13]. In Taiwan, not until the 1980s did several researchers start to research issues relevant to TCM, using sampling surveys or studies with small sample sizes [14-20]. To date, there has been no large-scale investigation of the use of TCM among Chinese people worldwide.

In Taiwan, the National Health Insurance (NHI) program was started in 1995 and covers nearly all inhabitants (21,653,555 beneficiaries at the end of 2001) [21-23]. The use of TCM has been reimbursed by the NHI since 1996. People in Taiwan are free to choose Western medicine or TCM, and are allowed to visit either public or private medical facilities. Because all claims data are available to researches in electronic form, we could conduct a study of TCM use among the Chinese population in Taiwan.

The aim of this study was to conduct a nation-wide survey in order to establish the frequency of TCM use, the characteristics of TCM users, and the medical conditions for which Taiwanese people most commonly use TCM, by analyzing the NHI claims data from 1996 to 2001. TCM provided by the NHI included Chinese herbal remedies, acupuncture and traumatology manipulative therapy [24].

## Methods

### Data Sources

The NHI program was initiated in Taiwan since 1995 and covers nearly all inhabitants (21,653,555 beneficiaries at the end of 2001, equivalent to a coverage rate of 96.6%). In 1999, the Bureau of NHI began to release all claims data in electronic form to the public under the National Health Insurance Research Database (NHIRD) project. The structure of the claim files is described in detail on the NHIRD website and in other publications [19,25].

We obtained the complete TCM claim datasets (CM_CD199601.DAT to CM_CD200112.DAT, 72 files) from the NHIRD in Taipei in November 2002. The datasets contained only the visit files, including dates, medical care facilities and specialties, patients' genders, dates of birth, and the three major diagnoses coded in the International Classification of Disease, 9^th ^Revision, Clinical Modification (ICD-9-CM) format [26-28]. To protect privacy, the data on patient identities and institutions had been scrambled cryptographically.

These visit files represented all the TCM outpatient activities within the NHI from 1996 to 2001. Insurance benefits were available for TCM that included Chinese herbal remedies, acupuncture and traumatology manipulative therapy, especially for joint dislocation. In Taiwan, TCM is reimbursed by NHI only in ambulatory clinics, not for inpatient care. In addition, only licensed TCM physicians qualify for reimbursement from the NHI. At the end of 2001, there were 2 public TCM hospitals, 42 private TCM hospitals and 2,544 private TCM clinics providing TCM ambulatory visits [22].

To calculate the numbers of valid beneficiaries in the study period, the beneficiaries' registry files (ID2002_1.DAT to ID2002_8.DAT) were also obtained.

### Study Design

Although the concept of disease entities in TCM is quite different from that in Western medicine, TCM physicians are requested to follow the standard diagnoses according to the ICD-9-CM coding system when claiming reimbursement. Common diagnostic groups for TCM visits were categorized according to the reclassification of primary ICD-9-CM codes for use in the National Ambulatory Medical Care Survey and National Hospital Ambulatory Medical Care Survey data in the United States [29].

To calculate patients' ages in relation to the 6-year use frequency of TCM from 1996 to 2001, December 31, 2001 was taken as the index of subtrahend. The denominator was the number of people who were insured during this 6-year period.

In order to compare the average numbers of visits between TCM and Western (allopathic) medicine, we also obtained the total number of ambulatory visits to Western medicine from the website of Department of Health, Taiwan [22]. In addition, we obtained the sampling claim datasets for ambulatory care visits at Western medicine clinics (S_CD 1996 to S_CD2001) in order to compare the top ten disease categories between TCM and Western medicine visits. The Western medicine files to be sampled were extracted from the complete outpatient claims (excluding dental and TCM services), using a sampling ratio of 0.2%. The sampling was random and visit-based but was separated monthly to eliminate possible seasonal variations. According to the NHIRD, these sampled files were representative of all utilisation within the NHI in Taiwan.

### Statistical Analysis

Microsoft SQL Server 2000 (MicroSoft Corp., Redmond, WA USA) was the main software used for data linkage and processing. Descriptive data, including frequencies, percentage and means, are presented.

## Results

Among the 21,653,555 valid beneficiaries of the NHI program at the end of 2001, 16,142,829 (28.4%) had used TCM during the year, but 13,536,266 (62.5%) had used TCM at least once during the whole 6-year period from 1996 to 2001, with a total of 156,224,266 visits (mean 11.5 visits per user). The annual number and percentage of TCM users steadily increased from 1996 to 2001 (Table 1). However, the annual number of TCM visits remained stationary from 1998 to 2001. There was a mean of 5,733,602 annual TCM users (ranging from 25.8% of the valid beneficiaries within NHI in 1996 to 28.4% in 2001) with a mean increment of 1,671,476 (29.2%) of new users yearly. Among TCM users, female was higher than male with a female:male ratio of 1.13:1. The age distribution of the TCM users peaked in the 30s, followed by the 20s and 40s (Table 2), while the age distribution for visit counts showed a peak in the 30s followed by the 40s and 20s. Adjusted for the total population in each 10-year age group, the use frequency of TCM still showed a peak in the 30s, followed by the 20s and 60s. When the details of each age group were investigated, it was found that more than 50% of patients used TCM if they were over 10 years old.

**Table 1:** Patient use and visit counts of traditional Chinese medicine (TCM) within National Health Insurance (NHI) from 1996 to 2001 in Taiwan

Year	Valid beneficiaries	Subjects using TCM			New patients	Total visits
	within NHI	Total No. (%)*	Female	Male	(%)	
1996	20,041,488	5,178,887 ^a ^(25.8%)	2,884,798 (55.7%)	2,276,795 (44.0%)	5,178,887 ^a^	24,094,552
1997	20,492,317	5,444,532 (26.6%)	3,025,170 (55.6%)	2,400,105 (44.1%)	2,506,004 ^b ^(46.0%)	25,170,073
1998	20,757,185	5,633,794 (27.1%)	3,135,701 (55.7%)	2,466,698 (43.8%)	1,862,599 ^c ^(33.1%)	26,641,926
1999	21,089,859	5,937,644 (28.2%)	3,305,115 (55.7%)	2,605,236 (43.9%)	1,556,868 ^d ^(26.2%)	27,398,595
2000	21,400,826	6,063,923 (28.3%)	3,384,892 (55.8%)	2,642,655 (43.6%)	1,314,192 ^e ^(21.7%)	26,426,753
2001	21,653,555	6,142,829 (28.4%)	3,420,592 (55.7%)	2,672,785 (43.5%)	1,117,716 ^f ^(18.2%)	26,492,367
Total			7,097,695^‡ ^(52.4%)	6,292,508^‡ ^(46.5%)	13,536,266^† ^(a+b+c+d+e+f)	156,224,266

* Number within parenthesis indicates percentage of subjects using TCM among valid beneficiaries within NHI in that year; ^†^146,063 people whose genders were not recorded; ^‡^Female:male = 1.13:1. New patients indicate people who used NHI-covered TCM for the first time.

**Table 2:** Age-specific usage frequency of traditional Chinese medicine (TCM) during the 6-year period from 1996 to 2001 in Taiwan

Age (years)	Number of total population*	Number of subjects using TCM (%)	Number of TCM visits
≤10	3,045,723	376,098 (12.3%)	3,585,106
11–20	3,384,508	2,087,947 (61.7%)	18,557,467
21–30	3,821,115	2,507,902 (65.6%)	22,970,206
31–40	3,802,330	2,509,366 (66.0%)	30,974,575
41–50	3,538,240	2,195,505 (62.1%)	29,908,451
51–60	2,051,260	1,209,673 (59.0%)	16,445,165
61–70	1,445,062	935,722 (64.8%)	13,234,416
71–80	989,984	580,621 (58.6%)	8,389,902
≥81	327,346	171,239 (52.3%)	2,084,788

*Total population number was obtained from Department of Internal Affairs, Executive Yuan, Taiwan, and data at the end of 2001 are presented.

**Figure 1:**
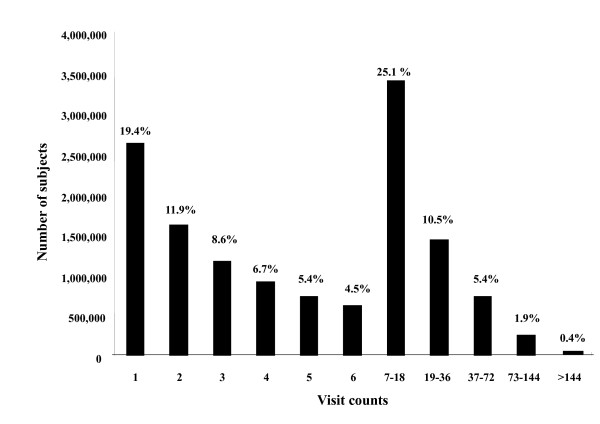
Visits counts per patient using traditional Chinese medicine during the 6 years from 1996 to 2001 in Taiwan.

Among the 13,536,266 subjects who experienced TCM during the 6-year period, 4,832,233 (35.7%) used TCM only in one year, 3,065,072 (22.6%) in two separate years, 2,106,523 (15.6%) in three separate years, 1,495,694 (11.0%) in 4 separate years, 1,083,577 (8.0%) in 5 separate years and 953,167 (7.0%) in all six years. As to the visit counts during this 6-year period, 19.4% of subjects used TCM only once, 47.8% used it more than 6 times (once per year on average) and 2.3% used it more than 72 times (once per month on average) (Figure 1).

Chinese herbal remedies (85.9%) were the most common TCM modality, followed by acupuncture (11.0%) and traumatology manipulative therapies (3.1%). Most of the TCM visits identified in the study were performed in private TCM clinics (82.6%), followed by private TCM hospitals (10.7%), others (4.7%, mostly Western medicine hospitals with TCM sections) and public TCM hospitals (0.7%). Visits to private TCM hospitals decreased yearly, while visits to private TCM clinics, public TCM hospitals and others increased (Table 3).

**Table 3:** Service volume of traditional Chinese medicine (TCM) by facility type from 1996 to 2001 in Taiwan

Year	Public TCM hospital	Private TCM hospital	Private TCM clinics	Others*
1996	79,817 (0.3%)	4,561,521 (18.9%)	18,612,160 (77.2%)	841,054 (3.5%)
1997	76,627 (0.3%)	3,845,787 (15.3%)	20,277,192 (80.6%)	970,467 (3.9%)
1998	226,207 (0.8%)	3,407,727 (12.8%)	21,963,148 (82.4%)	1,044,844 (3.9%)
1999	267,511 (1.0%)	2,889,723 (10.5%)	23,024,679 (84%)	1,216,682 (4.4%)
2000	280,086 (1.1%)	2,320,469 (8.8%)	22,371,552 (84.7%)	1,454,646 (5.5%)
2001	260,150 (1.0%)	1,766,232 (6.7%)	22,866,179 (86.3%)	1,599,806 (6.0%)
Total	1,190,398 (0.8%)	18,791,459 (12.0%)	129,114,910 (82.6%)	7,127,499 (4.6%)

*Others: mostly western medicine hospitals with TCM section

Among the 156,224,266 TCM visits, 150,365,543 (96.2%) had one clinical diagnosis, 4,955,213 (3.2%) had two diagnoses and 881,438 (0.6%) had three diagnoses according to the ICD-9-CM coding system. The top ten diseases for TCM visits were diseases of respiratory system; diseases of the musculoskeletal system and connective tissue; symptoms, signs and ill-defined conditions; injury and poisoning; diseases of the digestive system, the genitourinary system, the skin and subcutaneous tissue, the nervous system and sense organs, the circulatory and endocrine system; nutritional and metabolic diseases; and immunological disorders (Table 4). Common problems and diseases of major human organ systems recognised in Western medicine were all ranked in the top 41 diagnostic groups for TCM visits (Table 5). They included three disease groups relating only to females: disorders of menstration and abnormal bleeding (ICD code: 626.4), noninflammatory disorders of female genital organs (ICD code: 625.5) and inflammatory disorders of female pelvic organs (ICD code 614.9). Thus, people with diseases commonly seen in Western medicine used TCM.

**Table 4:** The top 10 major disease categories for traditional Chinese medicine visits from 1996 to 2001 in Taiwan

Major disease category	ICD-9-CM code	Number of visits	Percentage (%)
Diseases of the respiratory system	460–519	42,163,916	27.0
Diseases of the musculoskeletal system and connective tissue	710–739	25,922,217	16.6
Symptoms, signs and ill-defined conditions	780–799	22,273,597	14.3
Injury and poisoning	800–999	20,466,702	13.1
Diseases of digestive system	520–579	19,854,039	12.7
Diseases of the genitourinary system	580–629	11,269,623	7.2
Diseases of the skin and subcutaneous tissue	680–709	5,086,736	3.3
Diseases of the nervous system and sense organs	320–389	4,999,730	3.2
Diseases of the circulatory system	390–459	3,143,181	2.0
Endocrine, nutritional and metabolic disease and immune disorders	240–279	1,926,628	1.2

**Table 5:** The 41 most common diagnostic groups among 156,224,266 traditional Chinese medicine visits from 1996 to 2001 in Taiwan

Diseases diagnosis*	Number of visits	%
Other symptoms signs and ill-defined conditions	16,707,192	10.7
Other diseases of the digestive system	15,636,624	10.0
Acute sinusitis	13,790,219	8.8
Other diseases of the respiratory system	9,697,442	6.2
Other dorsopathies	9,593,900	6.1
Other injuries	8,265,712	5.3
Derangements and other unspecified joint disorders	6,219,704	4.0
Asthma	5,477,916	3.5
Other superficial injury	5,370,193	3.4
Other acute respiratory infections	4,741,441	3.0
Other rheumatism excluding back	4,702,518	3.0
Chronic sinusitis	4,553,347	2.9
Disorders of menstruation and abnormal bleeding	4,437,200	2.8
Other inflammatory conditions of skin and subcutaneous tissue	3,729,128	2.4
Contusions with intact skin surfaces	3,230,326	2.1
Non-inflammatory disorders of female genital organs	2,704,322	1.7
Ulcer of stomach and small intestine	2,181,074	1.4
Other disorders of the nervous system	2,063,541	1.3
Other disorders of the urinary system	1,786,787	1.1
Cough	1,723,945	1.1
Lumbago	1,524,536	1.0
Other sprains and strains	1,417,778	0.9
Allergic rhinitis	1,266,224	0.8
Other arthropathies and related disorders	1,167,430	0.7
Myalgia and myositis unspecified	1,167,300	0.7
Other mental disorders	1,127,697	0.7
Abnormal heart sounds	1,115,173	0.7
Acute bronchitis and bronchiolitis	1,069,862	0.7
Abdominal pain	1,051,691	0.7
Other diseases of the musculoskeletal system and connective tissue	1,039,678	0.7
Chronic and unspecified bronchitis	998,966	0.6
Gastritis and duodenitis	940,170	0.6
Inflammatory disorders of female pelvic organs	871,451	0.6
Other diseases of the circulatory system	844,021	0.5
Headache	825,747	0.5
Other diseases of the ear and mastoid process	817,140	0.5
Other disorders of the eye and adnexa	758,455	0.5
Other infections and parasitic diseases	746,719	0.5
Diabetes mellitus	740,528	0.5
Other diseases of central nervous system	731,867	0.5

* Only diseases with use frequency of traditional Chinese medicine greater than 0.5% are presented. Diagnostic groups were categorized according to the reclassification of the primary International Classification of Diseases diagnostic codes for use with National Ambulatory Medical Care Survey and National Hospital Ambulatory Medical Care Survey data in the United States.

Furthermore, we analyzed the percentage distribution of major disease categories for TCM visits by age (Table 6). The results show that for respiratory system diseases and injuries, the percentages of visits were higher in the younger groups and decreased with age. In contrast, for the categories of symptoms, signs and ill-defined conditions, musculoskeletal system, nervous system, circulatory system, endocrine and metabolic diseases, mental disorders and neoplasms, the percentages of visits were lower in the younger groups and increased with age. We found no significant differences between males and females in the percentage distributions of the commonest disease categories for TCM visits (Table 7). However, female subjects visited TCM for diseases of the genitourinary system (10.6%) more frequently than males (2.2%). We also compared the percentage distribution of major disease categories for TCM visits among different locations and the results revealed that the categories of injury, poisoning and symptoms, signs and ill-defined conditions were more commonly seen in clinics than in hospitals (Table 8).

**Table 6:** Percentage distribution of diseases categories for 156,224,266 traditional Chinese medicine visits by different age groups, 1996–2001, in Taiwan*

Major disease category	Years of age			
	0–19	20–39	40–59	>60
Symptoms, signs, ill-defined conditions^†^	13.2	13.2	15.0	16.2
Musculoskeletal system^†^	5.3	15.5	21.0	25.4
Nervous system and sense organs^†^	1.1	2.6	4.2	5.6
Circulatory system^†^	0.2	1.1	2.6	5.6
Endocrine and metabolic diseases^†^	0.2	0.8	1.8	2.3
Mental disorders^†^	0.2	0.7	1.0	1.0
Neoplasm^†^	0.1	0.2	0.4	0.4
Digestive system	9.5	12.9	13.9	13.6
Infectious and parasitic diseases	0.3	0.6	0.5	0.4
Genitourinary system	3.9	10.9	6.7	3.5
Respiratory system^‡^	48.3	22.6	20.4	18.6
Skin and subcutaneous tissue	4.0	4.3	2.2	1.7
Injury and poisoning	14.0	15.3	11.9	8.5

*5,836,651 (3.8%) visits had more than one diagnosis. ^†^Percentages increased with age. ^‡^Percentages decreased with age.

**Table 7:** Number of visits and percentage distribution of diseases categories for traditional Chinese medicine visits by gender, 1996–2001, in Taiwan

Major disease category	Female	Male
Diseases of the respiratory system	22486766 (24.7%)	19229835 (28.6%)
Diseases of the musculoskeletal system and connective tissue	14591874 (16.0%)	10960835 (16.3%)
Symptoms, signs and ill-defined conditions	13117731 (14.4%)	8962408 (13.4%)
Injury and poisoning	10681063 (11.7%)	9549648 (14.2%)
Diseases of the digestive system	10358168 (11.4%)	9254229 (13.8%)
Diseases of the genitourinary system	9637077 (10.6%)	1470300 (2.2%)
Diseases of the skin and subcutaneous tissue	3012960 (3.3%)	2021566 (3.0%)
Diseases of the nervous system and sense organs	2987998 (3.3%)	1973514 (2.9%)
Diseases of the circulatory system	1609018 (1.8%)	1496484 (2.2%)
Endocrine, nutritional and metabolic diseases and immunological disorders	934956 (1.0%)	954273 (1.4%)
Total	91204278	67149765

**Table 8:** Percentage distribution of diseases categories for 156,224,266 traditional Chinese medicine visits by location, 1996–2001, in Taiwan*

Major disease category	In Hospitals (%)	In Clinics (%)
Respiratory system	26.8	26.2
Musculoskeletal system	20.3	16.2
Digestive system	20.2	12.2
Genitourinary system	12.0	6.9
Symptoms, signs, ill-defined conditions	11.9^†^	14.3^†^
Nervous system and sense organs	6.5	3.0
Circulatory system	6.2	1.8
Skin and subcutaneous tissue	5.7	3.1
Endocrine and metabolic diseases	5.1	1.0
Injury and poisoning	4.8^†^	13.4^†^
Mental disorders	1.1	0.2
Neoplasm	1.7	0.2
Infectious and parasitic diseases	1.7	0.4

*5,836,651 (3.8%) visits had more than one diagnosis. ^†^Percentage in Clinics is higher than in Hospitals.

## Discussion

To the best of our knowledge, this study is the first extensive survey of TCM use in Chinese society. Only with the aid of a computerised insurance reimbursement database could such a large-scale TCM utilization study feasibly be analysed. Previous studies from western countries on the frequency and characteristics of CAM/TCM use have mainly consisted of surveys of clinic attendees, telephone interviews, written surveys, household interviews, and hospital and private clinic surveys; and the sample sizes have been limited. In addition, the use of CAM/TCM in western countries is usually not covered by insurance [30]. Thus, the survey results might be affected by the socio-economic status of the subjects [31]. Fortunately, TCM is reimbursed by NHI in Taiwan, so our study would appear to be less biased.

The use of CAM/TCM in western countries has increased dramatically in recent decades [1,5-8], [32-34]. It goes without saying that TCM has been commonly used in Asian countries, especially in the Chinese population, for centuries [17-19,35-37]. Owing to the different definitions of CAM, the types of CAM surveyed, survey methodologies and types of CAM reimbursed by insurance, it is difficult to compare the use frequency of CAM/TCM among countries [38,39]. According to our results, there was a steady increase in the annual number of TCM users in Taiwan between 1996 and 2001, and 62.5% of people used TCM covered by the NHI during this period; this does not include folk medicine, which is not reimbursable by insurance. The widespread use of TCM in the Chinese population might not be surprising since TCM has been developed in China for more than 2000 years and the ancestors of most Taiwanese were immigrants from China from the 17^th ^century onwards. Many concepts of TCM, such as the balance of Qi-blood, the regulation of body constitution and the mixture of herbs and food, have been part of Chinese culture and life style. Other ancient cultures in the world have similar experiences with their traditional medicine [40-42]. In addition, Chinese people believe that Western medicine may react faster to the target but also causes more adverse side effects, while TCM reacts slowly but is subtle and safe [16,43,44]. Furthermore, the insurance coverage for TCM visits might also play a significant role [31]. Lee et al. reported that TCM outpatient use rate increased 1.75-fold from 1983 to 1988 because of the opening of labour insurance coverage in Taiwan [14]. These factors might all account for the high utilization of TCM.

It is interesting to know how health care was used when both Western medicine and TCM were available in Taiwan. Table 9 compares the use frequencies of outpatient visits between TCM and Western medicine. The results show that people visited Western medicine clinics more commonly than TCM clinics for their illnesses. Thus, Western medicine remains the mainstream of health care. TCM outpatient visits accounted for around 9% of all outpatient clinics. Notably, the average number of outpatient visits per person per year in both TCM and Western medicine increased from 1996 to 1999, but decreased in 2000 owing to the rise in co-payments for outpatient visit under the NHI, Taiwan from August 1999 [22].

**Table 9:** Comparison of the average number of outpatient visits per person per year between Traditional Chinese Medicine (TCM) and Western Medicine (WM) from 1996 to 2001 in Taiwan.

Year	Valid beneficiaries	Total TCM visits	TCM visits per person	% change from previous year	Total WM visits	WM visits per person	% change from previous year
1996	20,041,488	24,094,552	1.20		221,566,817	11.06	
1997	20,492,317	25,170,073	1.23	2.17%	241,309,995	11.78	6.51%
1998	20,757,185	26,641,926	1.28	4.50%	259,701,792	12.51	6.25%
1999	21,089,859	27,398,595	1.30	1.22%	273,777,642	12.98	3.76%
2000	21,400,826	26,426,753	1.23	-4.95%	267,924,732	12.52	-3.56%
2001	21,653,555	26,492,367	1.22	-0.92%	267,412,756	12.35	-1.36%

Our finding that female use TCM more frequently than male, is consistent with previous reports from western countries [31,32,45,46]. Nevertheless, the reasons for this female predominance were not fully elucidated in previous reports. It was suggested that independent females, or females of good social status, had higher expectations of or belief in TCM in respect of postpartum conditions, menopause and chronic diseases [29-31], [47-49]. Our results show that disorders of menstruation and abnormal bleeding, noninflammatory disorders of female genital organs and inflammatory disorders of female pelvic organs were among the most common 40 disease groups for TCM visits, and this might in part account for the female predominance.

We found that the age distribution of TCM users peaked in the 30s, followed by the 20s and 40s. More than 50% of people over 10 years old had used TCM at least once in the 6 years surveyed. Previous studies have also shown that middle aged females are the characteristic CAM users in western countries [31,38,50,51]. A survey of German university hospitals found that young age was one of the predictors for a positive attitude towards CAM [52]. These results may indicate that adults are more frequent users of TCM/CAM than children [12][19].

Our results revealed that most TCM visits were to private TCM clinics (82.6%), followed by the private TCM hospitals (12.0%). This is supported by Chi's report, which showed that most of the active Chinese medicine physicians (82%) worked in personal practice clinics and only a small portion (18%) worked in Chinese medicine hospitals [53]. Also, in the United States, CAM (acupuncture) is available on a limited basis in major teaching hospitals [54]; and almost 40% of all general practices in Western medicine in the United Kingdom offer some form of access to CAM [38]. Further studies are needed to evaluate the role of TCM in both clinical practice and academic research in teaching hospitals.

According to our results, the most common reasons for TCM visits were diseases of the respiratory, musculoskeletal, digestive, genitourinary systems, and symptoms, signs and ill-defined conditions. Other common problems and diseases of major human organ systems recognised in Western medicine were all listed in the top 41 diagnostic groups for TCM visits. Our results are consistent with previous reports from western and Asian countries that various problems or diseases of human organ systems recognised in Western medicine were indicated for CAM use, including problems of the musculoskeletal, respiratory and digestive systems, neurological and psychological disorders, and general complaints [38,39,54-56]. It is also interesting to know for what kind of illnesses people in Taiwan seek help from TCM or Western medicine. Table 10 shows the top ten major disease categories for Western medicine outpatient visits from 1996 to 2001 in Taiwan, using 1:500 sampling from NHI files. The results show that all ten major disease categories were the same in both TCM and Western medicine, but the order from second to tenth was different. According to Linde, most people consult CAM for chronic pain resulting from chronic conditions or musculoskeletal system disorders [57]; and Chen et al. reported that more than 80% of indications for acupuncture visits in Taiwan were for musculoskeletal diseases [12]. These findings might explain the difference in the order of disease categories between TCM and Western medicine.

**Table 10:** Number and percentage distribution of visits to Western Medicine by major disease category from 1996–2001 in Taiwan.

Major disease category	ICD-9-CM code range	Visits	%
Diseases of the respiratory system	460–519	1,347,671	45.9%
Diseases of the digestive system	520–579	324,820	11.1%
Diseases of the nervous system and sense organs	320–389	297,257	10.1%
Diseases of the musculoskeletal system and connective tissue	710–739	251,625	8.6%
Diseases of the circulatory system	390–459	228,051	7.8%
Diseases of the genitourinary system	580–629	210,842	7.2%
Diseases of the skin and subcutaneous tissue	680–709	198,619	6.8%
Symptoms, signs, and ill-defined conditions	780–799	148,989	5.1%
Endocrine, nutritional and metabolic diseases and immunological disorders	240–279	138,771	4.7%
Injury and poisoning	800–999	97,843	3.3%
Total visits		2,935,480	100.0%

Data were obtained by 1:500 sampling from National Health Insurance files in Taiwan (see text for detail). All diagnoses at each claim were taken into consideration.

From the percentage distribution of diagnoses by age in our study, we found that for patients over the age of 20, conditions of major organ systems such as the musculoskeletal, nervous, circulatory and endocrine systems, as well as mental disorders, appeared more and more frequently in TCM visits, which might be a consequence of the aging process and the natural course of diseases. Evidently, respiratory conditions accounted for almost half of TCM visits in young people (aged under 20). Whether TCM has better efficacy and fewer side effects than Western medicine in treating conditions such as upper airway infection, asthma or allergy rhinitis deserves further evaluation [58,59]. We also found that TCM practitioners in clinics treated more injury conditions and symptoms, signs and ill-defined conditions than those in hospitals, while TCM practitioners in hospitals treated internal organ problems more than those in clinics [12]. Since the NHI program in Taiwan only covers outpatients, TCM practitioners in Chinese medicine sections in both Chinese and Western medical hospitals can deal with most ambulatory subjects, as in the clinics. However, in the hospitals, TCM doctors have to be trained strictly in either Chinese or Western medical school, unlike those in private clinics [53], and may have more diagnostic information from Western medicine, which may explain the greater number of visits for internal organ system conditions. We also found that female subjects visited TCM clinics for diseases of the genitourinary system more frequently than males. Our results are consistent with Foxman's report that women are significantly more likely than men to experience urinary tract infection [60]. In addition, the female use of TCM predominate for the genitourinary system was due to the disorders of menstruation and abnormal bleeding, noninflammatory disorders of female genital organs and inflammatory disorders of female pelvic organs were among the most common 40 disease groups for TCM visits.

In view of the substantially increased use of TCM/CAM, we suggest that medical doctors should ask patients about their use of TCM/CAM when taking a medical history. Exploration of the use of TCM/CAM will enhance the understanding of these practices and help patients and doctors to communicate during medical care. As Eisenberg and his colleagues suggested a decade ago, medical schools should design the curriculum to include information about CAM and clinical social sciences [2]. Several reports from western countries have concerned the need for CAM instruction to medical trainees and physicians [61,62]. Integration of Western medicine and TCM, in both medical education and clinical practice, should be initiated in countries where TCM and Western medication are widely used [53].

Our study has several limitations. First, NHI only reimburses Chinese herbal remedies in scientific granular or powder forms. Chinese herbal remedies in traditional herbal form such as Yin-Pian (prepared herbal medicine in small pieces ready for decoction, and medicine materials in crude slices) are not reimbursed and therefore are not included in our study. Secondly, our study did not include TCM visits provided by TCM clinics or hospitals that have no NHI contract, where patients need to pay entirely out-of-pocket. Finally, we did not include those Chinese herbal remedies obtained directly from traditional Chinese medicine pharmacies with or without prescriptions from licensed TCM doctors, nor did we include acupuncture performed by western-trained doctors. Thus, the use of TCM might have been underestimated in this study. Finally, without questionnaire surveys of patients, we were unable to ascertain the patients' beliefs, attitudes or inclinations towards TCM.

## Conclusion

TCM is popular in the Chinese population. More than 60% of subjects used TCM at least once during the 6-year study period. TCM, like western medicine, was commonly used by the Chinese population for problems and diseases of major human organ systems. Chinese herbal remedies were the most common TCM modality in Taiwan. This study provides information about the use frequencies of TCM and disease categories treated by TCM, which should be useful for health policy makers and for those who consider the integration of Chinese and Western medicine.

## Abbreviations

TCM: traditional Chinese medicine

CAM: complementary and alternative medicine

NHI: National Health Insurance

NHIRD: National Health Insurance Research Database

ICD-9-CM: International Classification of Diseases, Ninth Revision, Clinical Modification

## Competing interests

The author(s) declare that they have no competing interests.

## Authors' contributions

FPC conceived and carried out the study, performed the data analysis and drafted the manuscript. TJC and LFC participated in the design of the study and helped to perform the statistical analyses as well as to interpret findings. YCC, YYK and FJC performed the statistical analyses, helped to interpret findings and checked the grammar of the manuscript. SJH participated in the design and coordination of the study and helped to draft the manuscript. All authors read and approved the final manuscript.

## Pre-publication history

The pre-publication history for this paper can be accessed here:


